# Estrogen‐dependent gene regulation: Molecular basis of TIMP‐1 as a sex‐specific biomarker for acute lung injury

**DOI:** 10.14814/phy2.70047

**Published:** 2024-09-12

**Authors:** Sultan Almuntashiri, Saugata Dutta, Yin Zhu, Siddhika Gamare, Gustavo Ramírez, Valeria Irineo‐Moreno, Angel Camarena, Nora Regino, Paloma Campero, Carmen M. Hernández‐Cardenas, Tatiana S. Rodriguez‐ Reyna, Joaquin Zuñiga, Caroline A. Owen, Xiaoyun Wang, Duo Zhang

**Affiliations:** ^1^ Clinical and Experimental Therapeutics, College of Pharmacy University of Georgia and Charlie Norwood VA Medical Center Augusta Georgia USA; ^2^ Department of Clinical Pharmacy, College of Pharmacy University of Hail Hail Saudi Arabia; ^3^ Laboratory of Immunobiology and Genetics and Intensive Care Unit Instituto Nacional de Enfermedades Respiratorias Ismael Cosío Villegas Mexico City Mexico; ^4^ Tecnologico de Monterrey, School of Medicine and Health Sciences Mexico City Mexico; ^5^ Intensive Care Unit Instituto Nacional de Enfermedades Respiratorias Ismael Cosío Villegas Mexico City Mexico; ^6^ Department of Immunology and Rheumatology Instituto Nacional de Ciencias Médicas y Nutrición Salvador Zubirán Mexico City Mexico; ^7^ Division of Pulmonary and Critical Care Medicine Brigham and Women's Hospital, and Harvard Medical School Boston Massachusetts USA; ^8^ Department of Medicine, Medical College of Georgia Augusta University Augusta Georgia USA

**Keywords:** COVID‐19, fibroblasts, gene regulation, influenza A virus, PDGFRA, sex differences

## Abstract

Increased circulating tissue inhibitor of metalloproteinases‐1 (TIMP‐1) levels have been observed in patients with acute lung injury (ALI). However, the sex‐specific regulation of TIMP‐1 and the underlying molecular mechanisms have not been well elucidated. In this study, we found that plasma TIMP‐1 levels were significantly higher in COVID‐19 and H1N1 patients compared with those in healthy subjects (*n* = 25). TIMP‐1 concentrations were significantly different between males and females in each disease group. Among female but not male patients, TIMP‐1 levels significantly correlated with the PaO_2_/FiO_2_ ratio and hospital length of stay. Using the mouse model of ALI induced by the H1N1 virus, we found that TIMP‐1 is strikingly induced in PDGFRα‐positive cells in the murine lungs. Moreover, female mice showed a higher Timp‐1 expression in the lungs on day 3 postinfection. Mechanistically, we observed that estrogen can upregulate TIMP‐1 expression in lung fibroblasts, not epithelial cells. In addition, overexpression of estrogen receptor α (ERα) increased the *TIMP‐1* promoter activity. In summary, TIMP‐1 is an estrogen‐responsive gene, and its promoter activity is regulated by ERα. Circulating TIMP‐1 may serve as a sex‐specific marker, reflecting the severity and worst outcomes in female patients with SARS‐CoV2‐ and IAV‐related ALI.

## 1 INTRODUCTION

Severe acute respiratory syndrome coronavirus 2 (SARS‐CoV‐2) is the virus that causes coronavirus disease of 2019 (COVID‐19) (Cucinotta & Vanelli, 2020; Lu et al., 2020). The lung is the major organ targeted by COVID‐19. Lung inflammation induced by COVID‐19 is the primary cause of shortness of breath, respiratory failure, and acute respiratory distress syndrome (ARDS) in patients (Rodriguez‐Morales et al., 2020). Moreover, influenza viruses are also inflammatory lung pathogens that are known to cause seasonal epidemics and occasional pandemics (Taubenberger & Morens, 2008). Influenza A virus (IAV) is the most common and lethal among influenza viruses (Dangi & Jain, 2012). The severity of IAV can range from a mild upper respiratory tract infection to a severe lower respiratory tract infection that can lead to ARDS which is associated with high mortality (Kuiken et al., 2012).

Females have generally more robust immune responses than males (Klein & Flanagan, 2016). Females and males show differences in innate and adaptive immune responses to some viral infections (Furman et al., 2014; Jacobsen & Klein, 2021). Due to sex‐based differences in immune responses, sex has been investigated as a factor that may contribute to the morbidity and mortality of ARDS. In a large cohort of critically injured adults, women appeared to be more likely than men to develop ARDS, but no difference in mortality between the sexes was observed (Heffernan et al., 2011). Moreover, another cohort of patients with acute respiratory failure has reported that the female sex was significantly associated with higher mortality rates in patients with confirmed severe ARDS (McNicholas et al., 2019), although overall hospital mortality was not different in females and males. These studies indicated that sex hormones may either directly or indirectly contribute to increased lung injury and/or poorer clinical outcomes.

Estrogen is a steroid hormone responsible for developing female sexual traits and reproductive organs. Estrogen hormones are produced predominantly in the ovaries, corpus luteum, and placenta, but also can be produced by nongonadal organs like the liver. Currently, there are three major forms of estrogens in females namely estrone (E1), estradiol (E2), and estriol (E3). Each form of estrogen has a different role. E1 plays a major role after menopause, and it is synthesized from adrenal dehydroepiandrosterone in adipose tissue. E2 is the most potent estrogen during the premenopausal stage in a woman's life, while E3 plays an essential role during pregnancy, and it is produced in large amounts by the placenta (Cui et al., 2013).

Estrogen hormones have not been thoroughly investigated in inflammation and immune response. E1 has biological roles in the immune response by inducing NF‐κB‐driven inflammation in cancer cells (Diaz‐Ruano et al., 2023). E2 has been also reported to play a regulatory role in immune response, by not only inducing pro‐inflammatory cytokines but also by promoting macrophage activation (Liarte et al., 2011). Exogenous estradiol was also found to aggravate lung inflammation caused by *Pseudomonas aeruginosa* in a murine model of cystic fibrosis (Wang et al., 2010). A prospective clinical trial examined the predictive values of circulating sex hormone levels for 28‐day mortality and organ failure among septic shock patients (Feng et al., 2014). This study found that estradiol levels were significantly higher in non‐survivors than survivors and were independent predictors of mortality and acute kidney injury (Feng et al., 2014).

TIMPs (tissue inhibitor of metalloproteinases) regulate the enzymatic activity of matrix metalloproteinases (MMPs) and have a well‐recognized role in controlling extracellular matrix (ECM) turnover. *TIMP‐1* was the first‐identified natural collagenase inhibitor and has a genomic location on the X chromosome (Burkhardt et al., 2009). Different from other X‐linked genes, *TIMP‐1* is prone to reactivation and is variable in its inactivation (Anderson & Brown, 1999). Under inflammatory conditions, TIMP‐1 can be significantly induced by estradiol in goat oviductal epithelial cells and human aortic endothelial cells (Nasiri‐Ansari et al., 2022; Peng et al., 2015). Nevertheless, sex as a critical factor affecting TIMP‐1 expression has been ignored in prior TIMP‐1 studies. Our recent observational study in patients with ALI/ARDS showed that circulating TIMP‐1 levels are a promising predictor of mortality, ventilator‐free days, and ICU‐free days among females (Almuntashiri et al., 2022). Regarding MMPs, our team previously measured both MMP‐8 and MMP‐9 levels in plasma samples from IAV‐infected human subjects (Rojas‐Quintero et al., 2018). Neither MMP‐8 nor MMP‐9 levels showed a significant difference between the female and male groups. Furthermore, our team previously measured plasma MMP‐3 levels in ARDS patients enrolled in the ALTA trial (Jones et al., 2023). Different from MMP‐8 and MMP‐9, plasma MMP‐3 levels were higher in male patients, which is similar to the observation in patients with myocardial infarction (Trentini et al., 2022).

Our current study not only validated that sex‐biased circulating TIMP‐1 levels from COVID‐19 and H1N1 patients but also supported the potential of TIMP‐1 as a sex‐specific biomarker for female patients. Furthermore, we reported the PDGFRα‐expressing cells as the major source of increased TIMP‐1 in response to H1N1 infection and provided a molecular mechanism by which estrogen hormones regulate TIMP‐1 expression.

## 2 MATERIALS AND METHODS

### 2.1 Human plasma specimens, ethics approval, and plasma TIMP‐1 measurement

This is a secondary analysis using unidentifiable human plasma specimens. Augusta University Institutional Review Board has determined this project does not meet the definition of human subject research (IRB number: 2070085). Twenty‐five plasma samples from healthy subjects were purchased from Innovative Research (Novi, MI) to serve as controls. Sixty plasma samples from hospitalized patients with COVID‐19 were collected between March and October 2020. Eighty‐two plasma samples obtained from patients diagnosed with H1N1 IAV infection have been described in detail previously (Rojas‐Quintero et al., 2018). Plasma samples from patients were collected by the Institutional Review Boards at Instituto Nacional de Enfermedades Respiratorias Ismael Cosío Villegas, Mexico City. Informed consent was obtained from all participants. The authors assert that all procedures contributing to this work comply with the ethical standards of the relevant national and institutional committees on human experimentation and with the Helsinki Declaration of 1975, as revised in 2008. Human plasma TIMP‐1 levels were measured in duplicate using ELISA kit (DY970, R&D Systems, Minneapolis, MN).

### 2.2 Animal studies

The pandemic IAV strain, influenza A/California/07/2009 H1N1 (H1N1), was obtained from the Centers for Disease Control (CDC, Atlanta, GA), propagated in MDCK cells, and used to infect mice. Titers of stock were measured using a standard plaque assay on 6‐well plates of confluent monolayers of MDCK cells as we described previously (Rojas‐Quintero et al., 2018). Wild‐type (WT) C57BL/6 mice of both sexes (8–10 weeks old) were purchased from the Jackson Laboratory (Bar Harbor, ME). 1000 PFU was used to infect the mice. Mice were anesthetized with 2% isoflurane and inoculated by the intranasal route with a final volume of 50 μL (25 μL per nostril) of H1N1 in endotoxin‐free PBS or PBS alone as a control. On day 3 postinfection (p.i.), mice were euthanized using 5% isoflurane (Covetrus, Portland, ME). All the animal studies were approved by the Charlie Norwood Veterans Affairs Medical Center Institutional Animal Care and Use Committee and were following the ARRIVE guidelines.

### 2.3 Cell culture, treatment, transfection, and luciferase reporter assay

A594 (CCL‐185), BEAS‐2B (CRL‐3588), IMR‐90 (CCL‐186), MLg (CCL‐206), and HEK293T (CRL‐3216) cells were purchased from ATCC (Manassas, VA) and cultured according to the standard protocol. IMR‐90 and MLg were treated with 100 nM Estrone (E1) (HY‐B0234, MedChemExpress, Monmouth Junction, NJ) or Estradiol (E2) (HY‐B0141, MedChemExpress) for 6 or 24 h as indicated. Lipofectamine 2000 (11668019, Thermo Fisher Scientific) was used for transfection. At 48 h after transfection, a luciferase assay was performed with a Dual‐Luciferase Reporter Assay System (E1980, Promega, Madison, WI). Firefly and Renilla luciferase activities were measured using a GloMax luminometer (Promega).

### 2.4 RNA preparation, reverse transcription, and real‐time quantitative PCR (qPCR)

Total RNAs were isolated from cells using the RNAqueous Total RNA Isolation Kit (AM1914, Thermo Fisher Scientific). cDNA was generated by reverse transcription using the high‐capacity cDNA reverse transcription kit (4368814, Thermo Fisher Scientific). TBP was used as a reference gene. RT‐qPCR was performed on a StepOnePlus Real‐Time PCR System using the PowerUp SYBR Green Master Mix (A25742, Thermo Fisher Scientific). Primers were purchased from Integrated DNA Technologies. Sequences of primers are listed as follows:

human TBP: GATAAGAGAGCCACGAACCAC (forward) and CAAGAACTTAGCTGGAAAACCC (reverse); human TIMP‐1: GGAGAGTGTCTGCGGATACTTC (forward) and GCAGGTAGTGATGTGCAAGAGTC (reverse); mouse Tbp: TCAAACCCAGAATTGTTCTCC (forward) and GGGGTAGATGTTTTCAAATGC (reverse); mouse Timp‐1: GCAACTCGGACCTGGTCATAA (forward) and CGGCCCGTGATGAGAAACT (reverse).

### 2.5 Immunofluorescence staining and image acquisition

Formalin‐fixed cells or lung section were immunostained with anti‐mouse TIMP‐1 IgG (MA1‐773, Thermo Fisher Scientific) followed by goat anti‐mouse IgG conjugated to Alexa‐488 (A11003, Thermo Fisher Scientific). For double immunofluorescence staining, lung sections were immunostained with anti‐mouse TIMP‐1 IgG followed by goat anti‐mouse IgG conjugated to Alexa‐546 (A11001, Thermo Fisher Scientific). Lung sections were also immunostained with anti‐PDGFRα antibody (EPR22059‐270, Abcam, Cambridge, MA) followed by goat anti‐rabbit IgG conjugated to Alexa‐488 (A11070, Thermo Fisher Scientific). Isotype‐matched control mouse IgG (ab81216, Abcam) and rabbit IgG (ab37415, Abcam) were used as control primary antibodies. Cells or lung sections were counterstained with 4′,6‐diamidino‐2‐phenylindole (DAPI) (ab228549, Abcam, Cambridge, MA). All digital images were visualized at room temperature using a Zeiss Observer Z1 fluorescence (Carl Zeiss Microscopy), a Camera Axiocam 506 color camera (Carl Zeiss Microscopy), and the software Zeiss ZEN 3.0 pro edition (Carl Zeiss Microscopy) with a LD Plan‐Neofluar 40x/0.6 (Carl Zeiss Microscopy) objective lens.

### 2.6 Plasmids

Plasmids 3 × ERE luc and ERβ were gifts from Donald McDonnell (11354 and 22770, Addgene, Watertown, MA) (Hall & McDonnell, 1999; Wittmann et al., 2007). Plasmid ERα was a gift from Elizabeth Wilson (101141, Addgene) (Mao et al., 2008). Human *TIMP‐1* promoter fragments were cloned from human genomic DNA. The fragments of the *TIMP‐1* promoter were digested by Kpn I/Xho I and inserted into pGL3 basic luciferase reporter (E1751, Promega). Sequences of primers used for promoter cloning are listed as follows:

F1: CGGGGTACCAGGCCCAAGCACCTGGTATGCTGT (forward) and CCGCTCGAGCTGACAATGCAGGAACCTTCCCTG (reverse); F2: CGGGGTACCAGGCCCAAGCACCTGGTATGCTGT (forward) and CCGCTCGAGGCTGCGATTACATGCGTGAGCTAC (reverse); F3: CGGGGTACCGTAGCTCACGCATGTAATCGCAGC (forward) and CCGCTCGAGCTGACAATGCAGGAACCTTCCCTG (reverse).

### 2.7 Statistical analysis

Data were analyzed using SigmaPlot version 13 (Systat Software Incorporated, San Jose, CA). The Shapiro–Wilk test was used to determine whether the data were normally distributed, and Brown‐Forsythe test was used to assess equal variance. Normally distributed data are presented as mean ± SD. Data that were not normally distributed are presented as box‐plots showing medians and 25th and 75th percentiles, and whiskers showing min to max. One‐way ANOVAs followed by post hoc testing with 2‐sided Student's *t*‐tests or Mann–Whitney *U* tests were performed. *p* < 0.05 was considered statistically significant.

## 3 RESULTS

### 3.1 Study population

The demographic and summary characteristics of human subjects are shown in Table 1. There were no significant differences in the age between males and females within the healthy subject or H1N1 IAV‐infected groups. Among the patients with COVID‐19, females were older than males (*p* = 0.02) but had a significantly higher mean PaO_2_/FiO_2_ ratio (*p* = 0.009) and a significantly shorter mean hospital length of stay (*p* = 0.003). For patients infected with H1N1 IAV, females also had a significantly higher mean PaO_2_/FiO_2_ ratio (*p* = 0.03) but there was no significant difference in the mean hospital length of stay between the groups.

**TABLE 1:** Demographic and summary characteristics of subjects.

Healthy control				
Characteristic	All (*n* = 25)	Male (*n* = 7)	Female (*n* = 18)	*p* Value^a^
Age in years	40.36 (10.26)	38.57 (9.48)	41.06 (10.73)	0.57
PaO_2_/FiO_2_	–	–	–	–
Hospital stay in days	–	–	–	–

COVID‐19 patients				
Characteristic	All (*n* = 60)	Male (*n* = 30)	Female (*n* = 30)	*p* Value^a^
Age in years	50.30 (12.97)	46.43 (11.97)	54.17 (12.96)	0.02
PaO_2_/FiO_2_	200.40 (84.92)	173.45 (90.84)	227.34 (70.12)	0.009
Hospital stay in days	16.08 (15.56)	20.37 (17.25)	11.80 (12.53)	0.003

H1N1 IAV patients				
Characteristic	All (*n* = 82)	Male (*n* = 44)	Female (*n* = 38)	*p* Value^a^
Age (years)	47.84 (11.10)	46.64 (11.08)	49.24 (11.10)	0.34
PaO_2_/FiO_2_	117.83 (83.98)	105.63 (69.27)	133.62 (98.72)	0.03
Hospital stay in days	26.96 (18.04)	28.10 (11.82)	25.43 (24.28)	0.07

*Note*: Means and standard deviation (SD) were reported for continuous variables.

Abbreviations: FiO2, fraction of inspired oxygen; PaO_2_, partial pressure of oxygen.

^a^ Statistical test showing the comparison between male and female groups.

### 3.2 Plasma TIMP‐1 levels in healthy subjects and patients with COVID‐19

**FIGURE 1:**
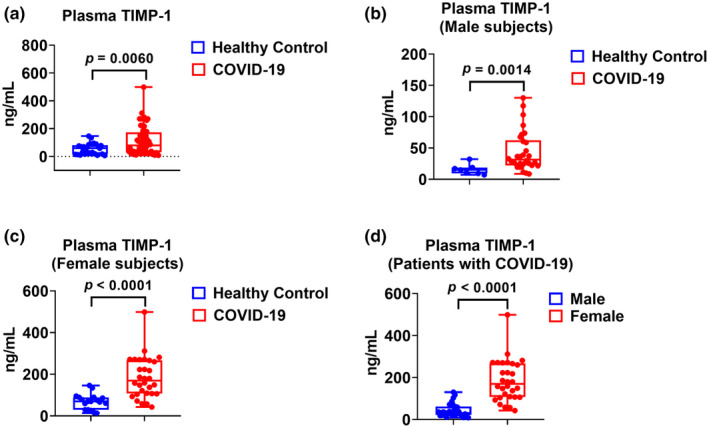
Plasma TIMP‐1 levels in patients with COVID‐19 and healthy subjects. (a) Plasma TIMP‐1 levels from healthy subjects (*n* = 25) and COVID‐19 patients (*n* = 60). (b) Plasma TIMP‐1 levels from male healthy subjects (*n* = 7) and male COVID‐19 patients (*n* = 30). (c) Plasma TIMP‐1 levels from female healthy subjects (*n* = 18) and female COVID‐19 patients (*n* = 30). (d) Comparison of plasma TIMP‐1 levels in female (*n* = 30) and male COVID‐19 patients (*n* = 30).

We measured plasma TIMP‐1 levels in both healthy subjects and patients with COVID‐19. Plasma TIMP‐1 levels were significantly higher in patients with COVID‐19 than in healthy subjects. The median level of TIMP‐1 was 59.51 ng/mL in the control group (*n* = 25) and 79.97 ng/mL in the COVID‐19 group (*n* = 60) (Figure 1a, *p* = 0.006 COVID‐19 vs. healthy). Among males, TIMP‐1 levels were also significantly higher in males with COVID‐19 than in healthy males. The median TIMP‐1 levels were 14.90 ng/mL in the control males (*n* = 7) and 31.36 ng/mL in males with COVID‐19 (*n* = 30) (Figure 1b, *p* = 0.0014 COVID‐19 males vs. healthy males). TIMP‐1 levels were much higher in females with COVID‐19 than in healthy females. The median TIMP‐1 level was 69.66 ng/mL in the control females (*n* = 18) and 169.1 ng/mL in females with COVID‐19 (*n* = 30) (Figure 1c, *p* < 0.0001 COVID‐19 females vs. healthy females). Lastly, TIMP‐1 levels were compared between female and male patients with COVID‐19, and females had significantly higher TIMP‐1 levels than males. The median level of TIMP‐1 was 31.36 ng/mL in the COVID‐19 males (*n* = 30) and 169.1 ng/mL in COVID‐19 females (*n* = 30) (Figure 1d, *p* < 0.0001 COVID‐19 males vs. females).

### 3.3 Plasma TIMP‐1 levels in healthy subjects and H1N1 IAV‐infected patients

**FIGURE 2:**
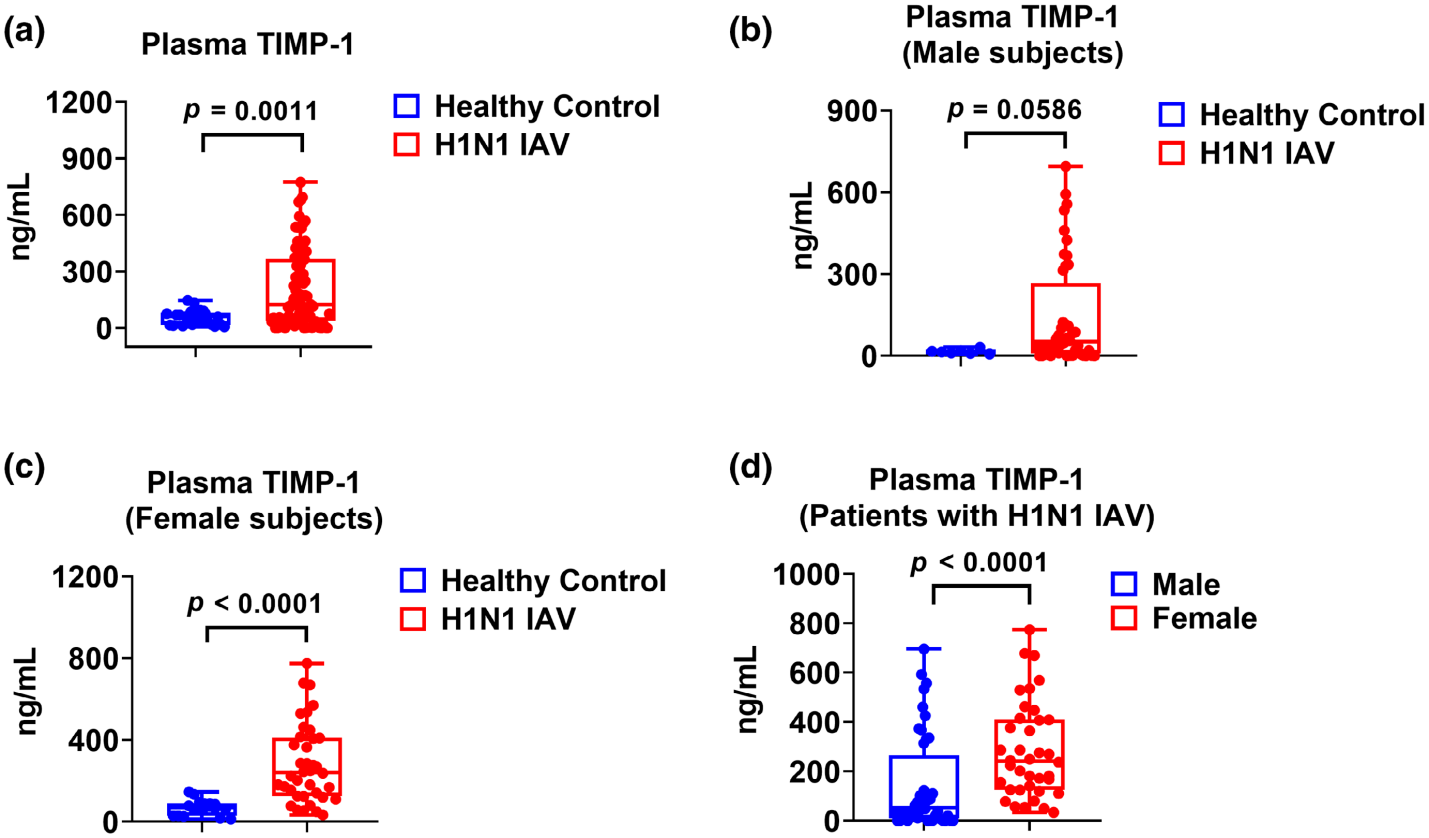
Plasma TIMP‐1 levels in patients with H1N1 and healthy subjects. (a) Plasma TIMP‐1 levels from healthy subjects (*n* = 25) and H1N1 patients (*n* = 82). (b) Plasma TIMP‐1 levels from male healthy subjects (*n* = 7) and male H1N1 patients (*n* = 44). (c) Plasma TIMP‐1 levels from female healthy subjects (*n* = 18) and female H1N1 patients (*n* = 38). (d) Comparison of plasma TIMP‐1 levels in males (*n* = 44) and female H1N1 (*n* = 38).

Next, we compared plasma TIMP‐1 in healthy subjects and H1N1 IAV‐infected patients. TIMP‐1 levels were significantly higher in H1N1 IAV‐infected patients than in healthy subjects. The median TIMP‐1 level was 59.51 ng/mL in the control group (*n* = 25) and 124.1 ng/mL in the H1N1 group (*n* = 82) (Figure 2a, *p* = 0.0011 H1N1 vs. healthy subjects). Among males, TIMP‐1 levels were not statistically significantly higher in H1N1 IAV‐infected males than in healthy males. The median level of TIMP‐1 was 14.90 ng/mL in the control males (*n* = 7) and 52.66 ng/mL in H1N1 males (*n* = 44) (Figure 2b, *p* = 0.0586 H1N1 males vs. healthy males). Among females, TIMP‐1 levels were significantly higher in H1N1 IAV‐infected females than in healthy females. The median level of TIMP‐1 was 69.66 ng/mL in the control females (*n* = 18) and 241.0 ng/mL in H1N1IAV‐infected females (*n* = 38) (Figure 2c, *p* < 0.0001 H1N1 females vs. healthy females). Next, plasma TIMP‐1 levels were compared between female and male H1N1 IAV‐infected patients, and levels were significantly higher in the females than the males. The median level of TIMP‐1 was 52.66 ng/mL in the H1N1 IAV‐infected males (*n* = 44) and 241.0 ng/mL in H1N1 IAV‐infected females (*n* = 38) (Figure 2d, *p* < 0.0001 H1N1 males vs. females).

### 3.4 Plasma TIMP‐1 level and association with relevant clinical parameters

**FIGURE 3:**
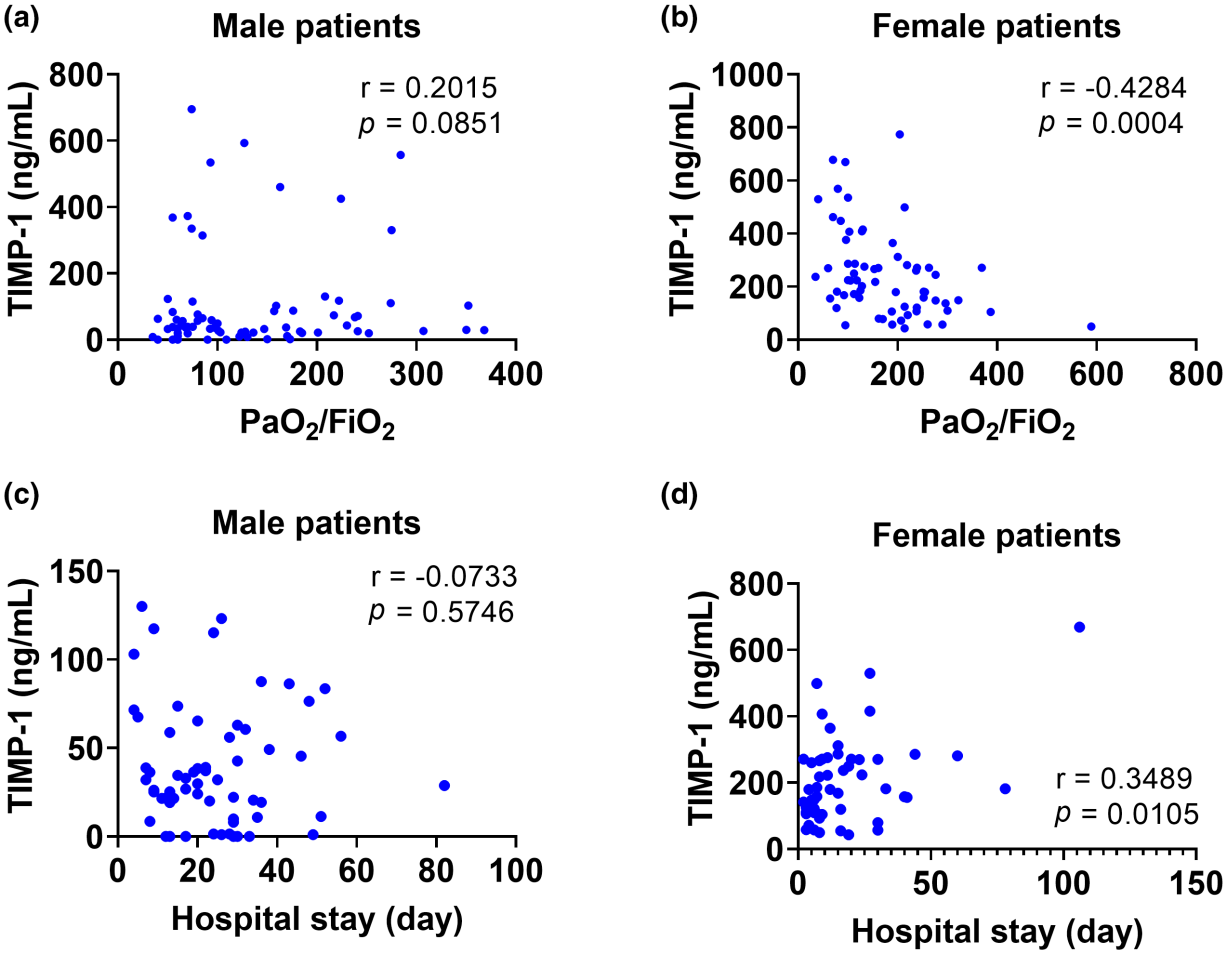
Correlation analyses between plasma TIMP‐1 and the clinical parameters in patients infected with either COVID‐19 or H1N1. Spearman correlation coefficient was used to test relationships between (a) TIMP‐1 and PaO_2_/FIO_2_ ratio; male patients (*n* = 74), (b) TIMP‐1 and PaO_2_/FIO_2_ ratio; female patients (*n* = 64), (c) TIMP‐1 and hospital length of stay; male patients (*n* = 61), (d) TIMP‐1 and hospital length of stay; female patients (*n* = 53).

We assessed whether TIMP‐1 plasma levels were related to relevant clinical parameters like PaO_2_/FiO_2_ ratio and hospital length of stay after combining patients infected with COVID‐19 and H1N1IAV. Among male patients, there was no significant correlation between TIMP‐1 plasma levels and the PaO_2_/FiO_2_ ratio (Figure 3a). Interestingly, plasma TIMP‐1 levels showed a significant negative correlation with the PaO_2_/FiO_2_ ratio among female patients. (Figure 3b, *r* = −0.4284, *p* = 0.0004). Among males, there was no significant correlation between plasma TIMP‐1 levels and hospital length of stay (Figure 3c). However, plasma TIMP‐1 levels showed a significant positive correlation with hospital length of stay among female patients. (Figure 3d, *r* = 0.3489, *p* = 0.0105).

### 3.5 Sex‐biased TIMP‐1 expression in murine lungs during H1N1 viral infection

**FIGURE 4:**
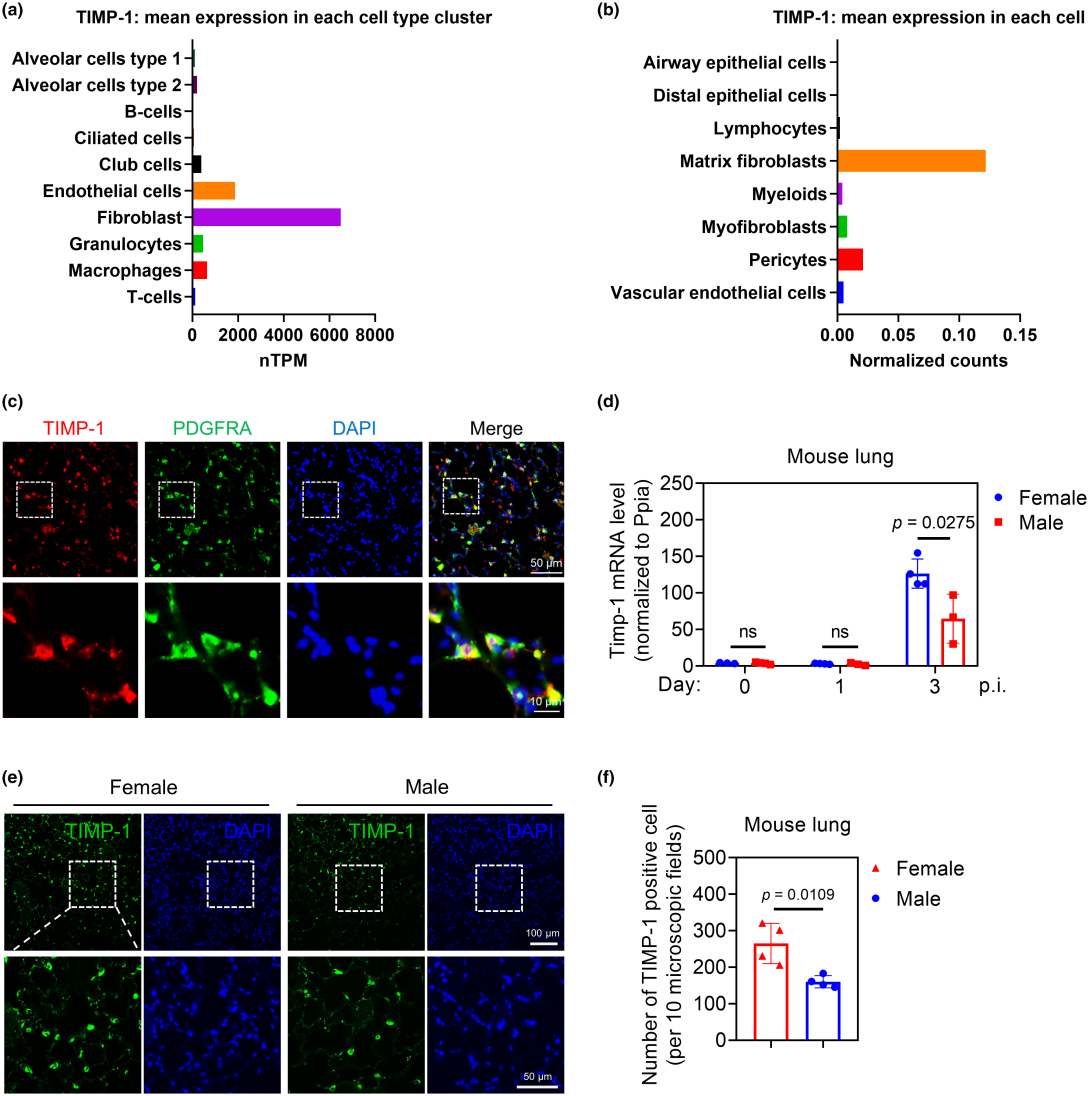
Sex‐biased TIMP‐1 expression in murine lungs during H1N1 viral infection. (a) The human lung single cell RNA‐sequencing data generated using the Human Protein Atlas database shows the relative TIMP‐1 mRNA expression in healthy lung tissues. (accessed on 07/30/2024). (b) The mouse lung single cell RNA‐sequencing data generated using Lung Gene Expression iN Single‐cell (LungGENS) database shows the relative TIMP‐1 mRNA expression in murine lungs at postnatal 28 days (accessed on 07/30/2024). (c) WT mice infected with 10^3^ PFU A/California/04/2009 H1N1 for 3 days (5–7 mice/group). Lung sections from H1N1‐infected WT mice on day 3 p.i. were immunostained for TIMP‐1 (red) and PDGFRα (green). The nuclei were stained with DAPI. Immunostained lung sections were examined with a confocal microscope and merged images. Images shown are representative of sections from 5 to 8 mice per experimental group. Scale bar = 50 μm and 10 μm as indicated. (d) Timp‐1 mRNA levels in female and male lungs (3–4 mice/group). (e and f) Lung sections from H1N1‐infected female and male mice (4 mice/group) on day 3 p.i. were immunostained for TIMP‐1 (green). The nuclei were stained with DAPI. Scale bar = 100 μm and 50 μm as indicated (e). TIMP‐1 positive cells were counted (f). ns, *p* > 0.05.

Single‐cell RNA sequencing analysis from the Human Protein Atlas (Figure 4a) and LungGENS (Figure 4b) database indicated that lung fibroblasts have the highest TIMP‐1 transcriptional levels among detected lung cells in both human and mice, respectively (Du et al., 2015; Karlsson et al., 2021). To locate cell type(s) that lead to the increase of TIMP‐1 in lung tissues after IAV infection, we infected C57BL/6 WT mice with 10^3^ plaque‐forming unit (PFU) A/California/04/2009 H1N1 IAV. The double immunofluorescence staining images showed the co‐localization of TIMP‐1 and PDGFRα in the murine lung section on day 3 p.i. (Figure 4c). It is known that PDGFRα is a marker of fibro‐adipogenic progenitor cells, which contributes to the rise of myofibroblasts and lipofibroblasts during lung development (Li et al., 2018; Trempus et al., 2023). The co‐localization indicated that PDGFRα‐positive fibroblasts are the main cellular source of the elevated TIMP‐1 during IAV infection. Next, we compared the Timp‐1 mRNA levels in the lungs of female and male mice. Interestingly, we found that female mice had higher Timp‐1 expression on day 3 p.i. (Figure 4d). The observation was confirmed using immunofluorescence staining (Figure 4e,f).

### 3.6 Estrogen induces TIMP‐1 expression in lung fibroblasts

**FIGURE 5:**
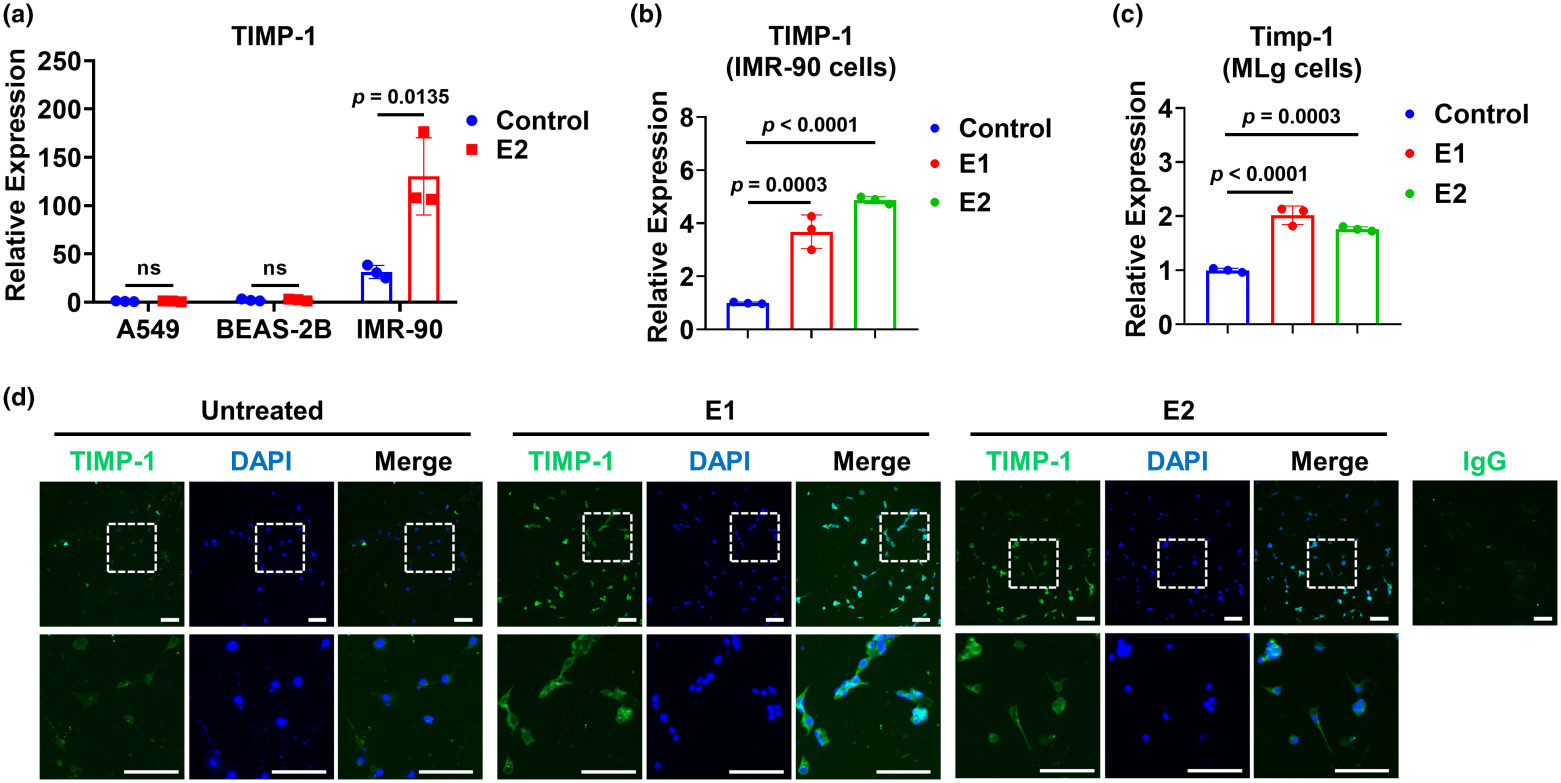
Estrogen induces TIMP‐1 expression in lung fibroblasts. (a) A549, BEAS‐2B, and IMR‐90 cells were incubated with 100 nM E2 for 6 h. TIMP‐1 mRNA expression was measured using qPCR. (B and C) IMR‐90 (b) and MLg cells (c) were treated with 100 nM E1 or E2 for 6 h. TIMP‐1 mRNA expression was measured using qPCR. (d) MLg cells were treated with or without 100 nM E1 or E2 for 24 h as indicated. MLg cells were immunostained for TIMP‐1 (green). The nuclei were stained with DAPI. Scale bar = 100 μm. ns, *p* > 0.05.

To test if estrogen can regulate TIMP‐1 expression, we treated human lung epithelial cells A549 and BEAS‐2B, as well as human lung fibroblasts IMR‐90 with estradiol E2. As shown in Figure 5a, E2 upregulated TIMP‐1 expression in fibroblasts IMR‐90 but not in epithelial cells A549 or BEAS‐2B cells. In addition, we found that both E1 and E2 can induce TIMP‐1 expression in IMR‐90 cells at the mRNA level (Figure 5b). A similar observation was found in murine lung fibroblasts MLg (Figure 5c). In addition, we measured TIMP‐1 protein levels in the cells using immunofluorescence staining. TIMP‐1 protein was induced in MLg fibroblasts 24 h after E1 or E2 treatment (Figure 5d).

### 3.7 ERα regulates TIMP‐1 promoter activity

**FIGURE 6:**
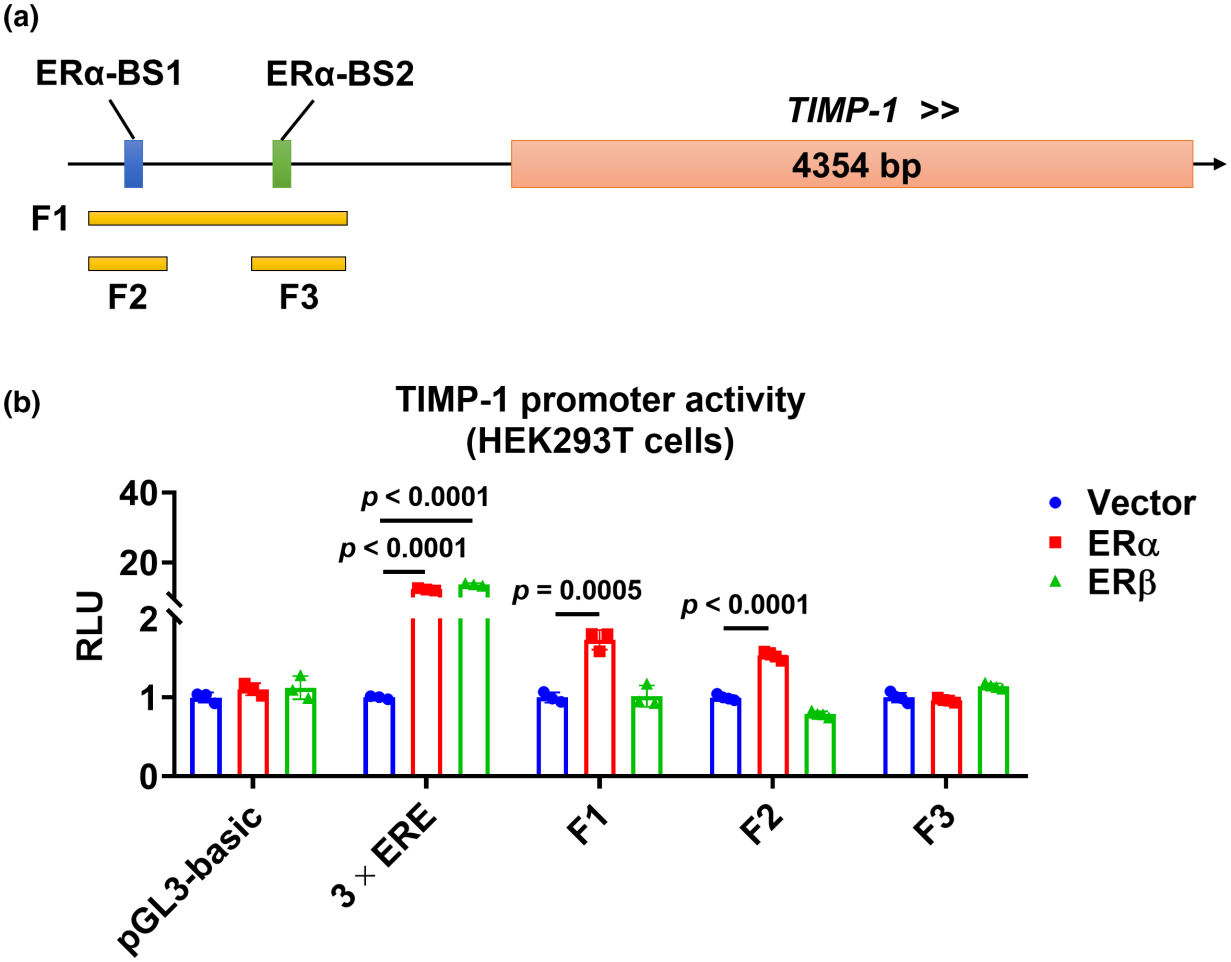
ERα regulates TIMP‐1 promoter activity. (a) Predicted ERα binding sites in the human *TIMP‐1* promoter region. Fragments (F1–F3) were subcloned into a luciferase reporter. (b) The luciferase activity containing F1, F2, or F3 was measured using HEK293T 48 h after they were co‐transfected with ERα or ERβ as indicated. The pGL‐3basic luciferase reporter (Promega) empty backbone plasmid was used as a negative control. Luciferase reporter containing three copies of *vitellogenin* estrogen response element (3 × ERE) was used as a positive control.

As shown in Figure 6a, we identified two ERα binding sites, ERα binding site 1 (ERα‐BS1) and ERα binding site 2 (ERα‐BS2), in the human *TIMP‐1* promoter using the UCSC Genome Browser. Interestingly, a luciferase reporter assay showed that the fragments (F1 and F2) that contain the ERα‐BS1 were subcloned into the luciferase reporter, increased luciferase activity was detected when they were co‐transfected into cells with ERα, but not ERβ (Figure 6b). When fragment F3 containing the ERα‐BS2 was subcloned into the luciferase reporter, no increase in luciferase activity was detected when they were co‐transfected with either ERα or ERβ. Taken together, the data indicate *TIMP‐1* is an estrogen‐responsive gene with an ERα binding site in its promoter.

## 4 DISCUSSION

Cytokines, chemokines, growth factors, and pharmacologic agonists have been shown to activate TIMP‐1 expression (Almuntashiri et al., 2023). Among these known modulators and pathways, TGF‐β/Smad is a well‐known upstream signaling of TIMP‐1 (Bonniaud et al., 2004; Leivonen et al., 2013), particularly in fibroblasts. Recombinant TGF‐β1 induced‐TIMP‐1 expression was observed in WT fibroblasts but not in *Smad3* knockout (KO) fibroblasts (Bonniaud et al., 2004; Leivonen et al., 2013). Although the regulation of TIMP‐1 has been extensively studied, no prior study has evaluated the female sex as a factor that could significantly affect TIMP‐1 expression under physiological or pathological conditions. In addition, elevated circulating TIMP‐1 levels have been shown to correlate with the severity of ALI/ARDS (Almuntashiri et al., 2023). However, the cells that contribute to the increased TIMP‐1 is unclear.

TIMP‐1 expression is strongly induced during ALI and a previous study reported that loss of *Timp‐1* had protective effects by reducing lung inflammation (Almuntashiri et al., 2023). In a prior study of a large cohort of mechanically ventilated critically ill patients and a subgroup of ARDS patients, high plasma TIMP‐1 levels were associated with severe hypoxemia and increased mortality (Hastbacka et al., 2014). In preclinical studies, *Timp‐1* deficient mice exhibited less body weight loss than WT mice after *Pseudomonas aeruginosa* (Lee et al., 2005) or influenza infection (Allen et al., 2018). *Timp‐1*‐deficient mice also showed fewer immune cell infiltrates and lung inflammation after influenza infection (Allen et al., 2018). The knockdown of TIMP‐1 using siRNA also reduced lung inflammations in an animal model of lipopolysaccharide (LPS)‐induced ALI (Chernikov et al., 2023). On the other side, TIMP‐1 plays a critical role in ECM turnover and lung remodeling during the development of pulmonary diseases (Arpino et al., 2015). TIMP‐1 overexpression by gene transfer can aggravate hypoxia‐induced pulmonary hypertension (Vieillard‐Baron et al., 2000). Studies also indicate that TIMP‐1 involves in the pathogenesis of pulmonary fibrosis (Madtes et al., 2001; Manoury et al., 2006). Therefore, factors that affect TIMP‐1 expression warrant additional studies in diseases associated with lung inflammation and remodeling.

Biological sex has been reported to influence susceptibility to infection, immune response, disease severity, and response to therapy (Dias et al., 2022; Giefing‐Kroll et al., 2015). Likewise, susceptibility to symptomatic COVID‐19 appeared to be associated with several variables including biological sex (Klein et al., 2020). A large US‐based cohort study indicates that male sex is a potential risk factor for higher morbidity and mortality from COVID‐19 (Kharroubi & Diab‐El‐Harake, 2022). El Aidaoui et al., 2022 reported that COVID‐19 gender disparities might be due to sex differences in the immune response including the regulatory roles of sex hormones and sex chromosomes genes (El Aidaoui et al., 2022). In a prospective cohort study, the female gender was associated with long COVID syndrome as defined by the continuation of symptoms for weeks after recovery (Bai et al., 2022). When exposed to IAV, females had a higher fatality rate than males (Jacobs et al., 2012; World Health Organization, 2021. Sex, gender and influenza. https://apps.who.int/iris/handle/10665/44401), and females had higher hospitalization rates than males (53.2% vs. 46.8%, respectively) during the 2009 H1N1 pandemic (World Health Organization, 2021. Sex, gender and influenza. https://apps.who.int/iris/handle/10665/44401). Sex differences in the severity of IAV infection can be recapitulated in other mammals suggesting that biological differences between the sexes may be involved (Humeniuk et al., 2023). However, the pathways and molecular mechanisms that mediate sex differences in response to COVID‐19 and IAV infection have not been well elucidated.

Estrogens exert their activities by binding to the estrogen receptors, which in turn trigger gene transcription and signaling cascades leading to the activation of downstream pathways (Eyster, 2016; Fuentes & Silveyra, 2019). Robinson et al demonstrated that estradiol (E2) is an anti‐inflammatory hormone and reduces the severity of influenza A virus infection in females (Robinson et al., 2014). Estrogen receptors are not only located in the female reproductive system and breast but also in other tissues and organs including bone, skin, brain, liver, lung, colon, and salivary gland. Two nuclear estrogen receptors (ERα and ERβ) and one membrane estrogen receptor (GPER1) have been described so far (Eyster, 2016; Fuentes & Silveyra, 2019). In asthma‐related airway inflammation, ERβ was upregulated in human airway smooth muscle cells (Aravamudan et al., 2017), and *ERβ*‐deficient mice showed an exacerbated airway hyperresponsiveness in response to allergen‐induced asthma (Kalidhindi et al., 2019). *ERβ*‐deficient mice also show abnormalities in alveolar structure and extracellular matrix protein composition (Morani et al., 2006). Increased levels of the estrogen receptors, ERα and ERβ, were reported in a murine model of LPS‐induced ALI (Jia et al., 2015). In the current study, we found that ERα (but not Erβ) modulates TIMP‐1 promoter activity. Interestingly, in a murine model of carrageenan‐induced pulmonary inflammation, E2 has anti‐inflammatory activities that are mediated via ERα but not ERβ (Vegeto et al., 2010). To confirm that ERα regulates TIMP‐1 expression, our future studies will compare the TIMP‐1 expression in WT versus *ERα*‐deficient mice in the unchallenged state and murine models of ALI.

There are several limitations in our study. The first issue comes from the small sample sizes and differences in demographic data (e.g., age) between males and females in some of the groups. The sample sizes were insufficient to evaluate whether plasma TIMP‐1 levels were related to ventilator‐free days, ICU‐free days, mortality, or other key clinical outcomes in patients infected with SARS‐CoV2 or H1N1 IAV. Second, this is a retrospective study with notable disadvantages in experimental design. For instance, we were unable to measure the kinetics of circulating TIMP‐1 in different stages of the infection, which is critical for the potential use of TIMP‐1 as a prognostic biomarker. In addition, although two independent cohorts were analyzed, confounding variables such as medications and other comorbidities were not considered, which may affect the TIMP‐1 levels in the circulation. Moreover, we did not perform a correlation analysis between TIMP‐1 and other inflammatory mediators. It is known that inflammatory mediators, such as TNF‐α and IL‐1β, can regulate TIMP‐1 expression (Almuntashiri et al., 2023). Therefore, further studies are needed to determine whether inflammatory mediators participate in the sex‐specific regulation of TIMP‐1 during lung infections. Finally, we were unable to determine whether TIMP‐1 is a significant downstream mediator of the effects of estrogen in the immune response following viral infections in humans or the mechanisms by which higher plasma TIMP‐1 levels in females may contribute to severe hypoxemia as assessed by higher PaO_2_/FiO_2_ ratios or prolonged length of hospital stays when compared to outcomes in males following SARS‐CoV2 and/or H1N1 IAV infections. Thus, our future studies will also address whether TIMP‐1 has an immunoregulatory in WT versus *Timp‐1*‐deficient mice in murine models of COVID‐19 or IAV infection. Sex differences between males and females will be also considered. Nevertheless, our study shows for the first time that *TIMP‐1* is an estrogen‐responsive gene.

## AUTHOR CONTRIBUTIONS

XW and DZ conceived the project and designed experimental protocols. SA, SD, YZ, and SG acquired data and analyzed and interpreted results. JZ, GR, VI, AC, NR, PC, CMH, and TSR collected clinical samples and information. SA, CAO, XW, and DZ wrote the manuscript. All authors reviewed and approved the manuscript.

## FUNDING INFORMATION

This work was supported by National Institute of Allergy and Infectious Diseases (NIAID) grant R03 AI169063 and NIH‐funded Georgia CTSA KL2 and UL1 grants (KL2TR002381 and UL1TR002378) to XW; National Heart, Lung, and Blood Institute (NHLBI) grant R00HL141685 and R56HL163607 to DZ. The content is solely the responsibility of the authors and does not necessarily represent the official views of the National Institutes of Health.

## CONFLICT OF INTEREST STATEMENT

The authors have declared that no conflict of interest exists. CAO is a current employee of AstraZeneca biopharmaceuticals R&D and may own shares in this company. CAO has declared that no conflict of interest relevant to this manuscript.

## ETHICS STATEMENT

This is a secondary analysis using unidentifiable human plasma specimens and determined as non‐human subject research. The study is approved by Augusta University Institutional Review Board (“Decoding the Molecular Mechanisms of Lung Diseases”, IRB number: 2070085, initial approval date June 20, 2023). All research procedures were in accordance with the ethical standards of the Augusta University Institutional Review Board and with the amended Helsinki Declaration of 1975.

## Data Availability

The datasets used and/or analyzed during the present study are available from the corresponding author upon reasonable request.
